# Periconception onset diabetes is associated with embryopathy and fetal growth retardation, reproductive tract hyperglycosylation and impaired immune adaptation to pregnancy

**DOI:** 10.1038/s41598-018-19263-8

**Published:** 2018-02-01

**Authors:** Hannah M. Brown, Ella S. Green, Tiffany C. Y. Tan, Macarena B. Gonzalez, Alice R. Rumbold, M. Louise Hull, Robert J. Norman, Nicolle H. Packer, Sarah A. Robertson, Jeremy G. Thompson

**Affiliations:** 1 Robinson Research Institute, Adelaide Medical School, University of Adelaide, Adelaide, Australia; 2 Australian Research Council (ARC) Centre for Nanoscale Biophotonics, University of Adelaide, Adelaide, Australia; 3 Fertility SA, Adelaide, Australia; 4 ARC Centre for Nanoscale Biophotonics, Department of Molecular Sciences, Macquarie University, Sydney, Australia

## Abstract

Diabetes has been linked with impaired fertility but the underlying mechanisms are not well defined. Here we use a streptozotocin-induced diabetes mouse model to investigate the cellular and biochemical changes in conceptus and maternal tissues that accompany hyperglycaemia. We report that streptozotocin treatment before conception induces profound intra-cellular protein β-*O*-glycosylation (*O*-GlcNAc) in the oviduct and uterine epithelium, prominent in early pregnancy. Diabetic mice have impaired blastocyst development and reduced embryo implantation rates, and delayed mid-gestation growth and development. Peri-conception changes are accompanied by increased expression of pro-inflammatory cytokine *Trail*, and a trend towards increased *Il1a*, *Tnf and Ifng* in the uterus, and changes in local T-cell dynamics that skew the adaptive immune response to pregnancy, resulting in 60% fewer anti-inflammatory regulatory T-cells within the uterus-draining lymph nodes. Activation of the heat shock chaperones, a mechanism for stress deflection, was evident in the reproductive tract. Additionally, we show that the embryo exhibits elevated hyper-*O*-GlcNAcylation of both cytoplasmic and nuclear proteins, associated with activation of DNA damage (ɣH2AX) pathways. These results advance understanding of the impact of peri-conception diabetes, and provide a foundation for designing interventions to support healthy conception without propagation of disease legacy to offspring.

## Introduction

Worldwide, one in seven pregnancies are complicated by diabetes^1^. Pregnancy in women with diabetes is associated with increased risk of fetal, neonatal and obstetric complications, maternal morbidity and mortality, and a 4- to 10-fold elevated risk that infants will develop diabetes as adults^2^. Adverse outcomes include fetal and neonatal death, congenital abnormalities, premature delivery and macrosomia, associated with an array of obstetric complications including but not limited to birth trauma, stillbirth, respiratory distress syndrome and neonatal hypoglycaemia. Maternal complications include increased risk of preeclampsia, caesarean section, worsening of pre-existing diabetic complications, and in rare cases, maternal death^2,3^. The peri-conception phase of early pregnancy is essential for setting in train fetal development and offspring phenotypes, and perturbations at this time can impart an elevated diabetes risk to offspring. In diabetic women, pre-conception treatment to control hyperglycaemia significantly reduces the risk of diabetic-induced pregnancy complications, showing the adverse effects are largely due to dysregulated glucose metabolism^4–6^. However if glycaemic control was achieved in the first weeks of pregnancy but not until after the peri-conception window, there was substantially less benefit afforded to the risk of pregnancy loss or incidence of fetal abnormality, implying excessive glucose is most detrimental in the conception and implantation phase^7,8^.

Animal studies allow detailed investigation of hyperglycaemia during the peri-conception period and provide evidence of disadvantage to offspring. Remarkably, a single day’s exposure of a pre-implantation embryo to the reproductive tract of a diabetic mouse, followed by transfer to a pseudo-pregnant non-diabetic recipient, results in substantial fetal growth and congenital abnormalities^9^ and transmission of diabetes to the offspring^10^. Many of the detrimental effects of high glucose bioavailability in hyperglycaemia are mediated by increased flux through the hexosamine biosynthetic pathway (HBP). A major endpoint of the HBP is the formation of uridine diphosphate β-D-N-acetylglucosamine (UDP-GlcNAc), the donor for nuclear and cytoplasmic protein β-*O*-linked glycosylation (*O*-GlcNAc), and complex extracellular N- and *O*-linked membrane protein glycosylation. *O*-GlcNAc is a dynamic nutrient-sensitive post-translational modification characterized by the addition of single N-acetylglucosamine to serines and/or threonines of almost every functional class of intracellular protein, which cycles on and off proteins due to the enzymatic action of a single transferase (beta-O-linked N-acetylglucosamine transferase; OGT) and a hydrolase (beta-N-acetylglucosaminidase; OGA). *O*-GlcNAc serves as a nutrient/stress sensor regulating several cellular processes, such as signalling, transcription, cytoskeletal dynamics, and cell division^11^. Altered *O*-GlcNAc signalling is directly involved in the pathogenesis of diabetes and new insights are revealing the importance of *O*-GlcNAc in diabetic complications^12^. It is not known whether *O*-GlcNAc is altered within the reproductive tract in diabetes, whether this is evident during early development, and if maternal hyperglycemia influences *O*-GlcNAc in embryos.

During *in vitro* development, oocytes and zygotes cultured briefly in the absence of glucose are unable to complete embryo compaction, failing to progress beyond the morula stage^13^. Hyperglycaemic culture conditions are also toxic to embryos^14^, indicating that normal development requires a narrow glucose concentration range. Our previous *in vitro* studies demonstrate the importance of peri-conception hyperglycemic control for normal development and identified *O*-GlcNAc as a key regulator^14–17^. Notably, exposure to hyperglycaemia caused altered stress deflection in the cumulus oocyte complex via aberrant *O*-GlcNAc glycosylation of the heat shock chaperones, which prevented normal preimplantation embryo development^15^.

There is a pressing need to address the mechanisms by which a diabetic environment *in utero* alters the trajectory of offspring development in order to devise interventions to mitigate the increased risk of fetal, neonatal and obstetric disorders in pregnancies complicated by diabetes^2^ and to reduce disease risks for children born to diabetic mothers. We hypothesised that diabetes during the periconception period acts to alter *O*-GlcNAc abundance and stress deflection pathways important for early development. Furthermore, given the well-described role of *O*-GlcNAc in immune cell regulation and cytokine signalling^18^, the impact of hyperglycaemia on inflammatory parameters^19^, and the significance of the maternal immune response for fetal growth and offspring health^20^, we predicted a role for the local and systemic immune milieu in mediating the adverse effect of diabetes on early development. Here we utilise the well-defined streptozotocin-induced diabetes mouse model to define the impact of periconception-onset diabetes on biochemical and inflammatory parameters in the reproductive tract, and the consequences for early embryo development and pregnancy progression.

## Results

### Periconception-onset diabetes impairs pre-implantation embryo development, and increases DNA damage and protein *O*-GlcNAcylation

**Figure 1:**
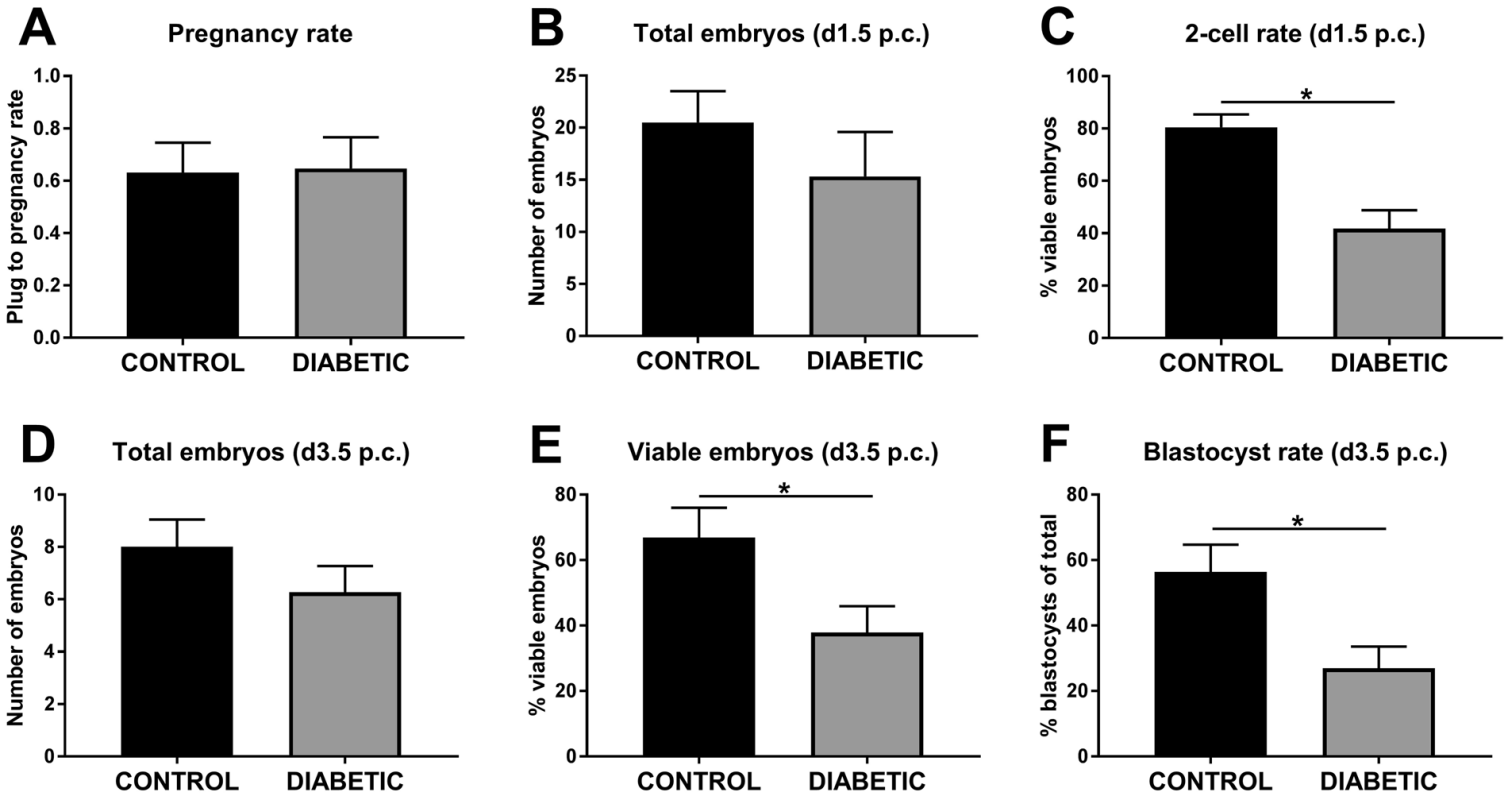
Pre-gestational diabetes significantly impairs preimplantation embryo viability. Control (black) and diabetic (grey) female mice were hormone-stimulated and mated, then embryos were flushed from the oviduct on d1.5 p.c (**A**–**C**) and uterus on d3.5 p.c (**D**–**F**). Conception rate (proportion of females with at least one viable embryo following mating, **A**), total 2-cell embryo number (**B**), % viable 2-cells (**C**), total embryos on d3.5 p.c. (**D**), viable embryos on d3.5p.c. (**E**) and blastocyst rate (**F**) were assessed (n = 13 females mated per treatment, per time point). Data is presented as mean + SEM and effects of treatment were analysed by unpaired Student’s t-test. *P < 0.05.

**Figure 2:**
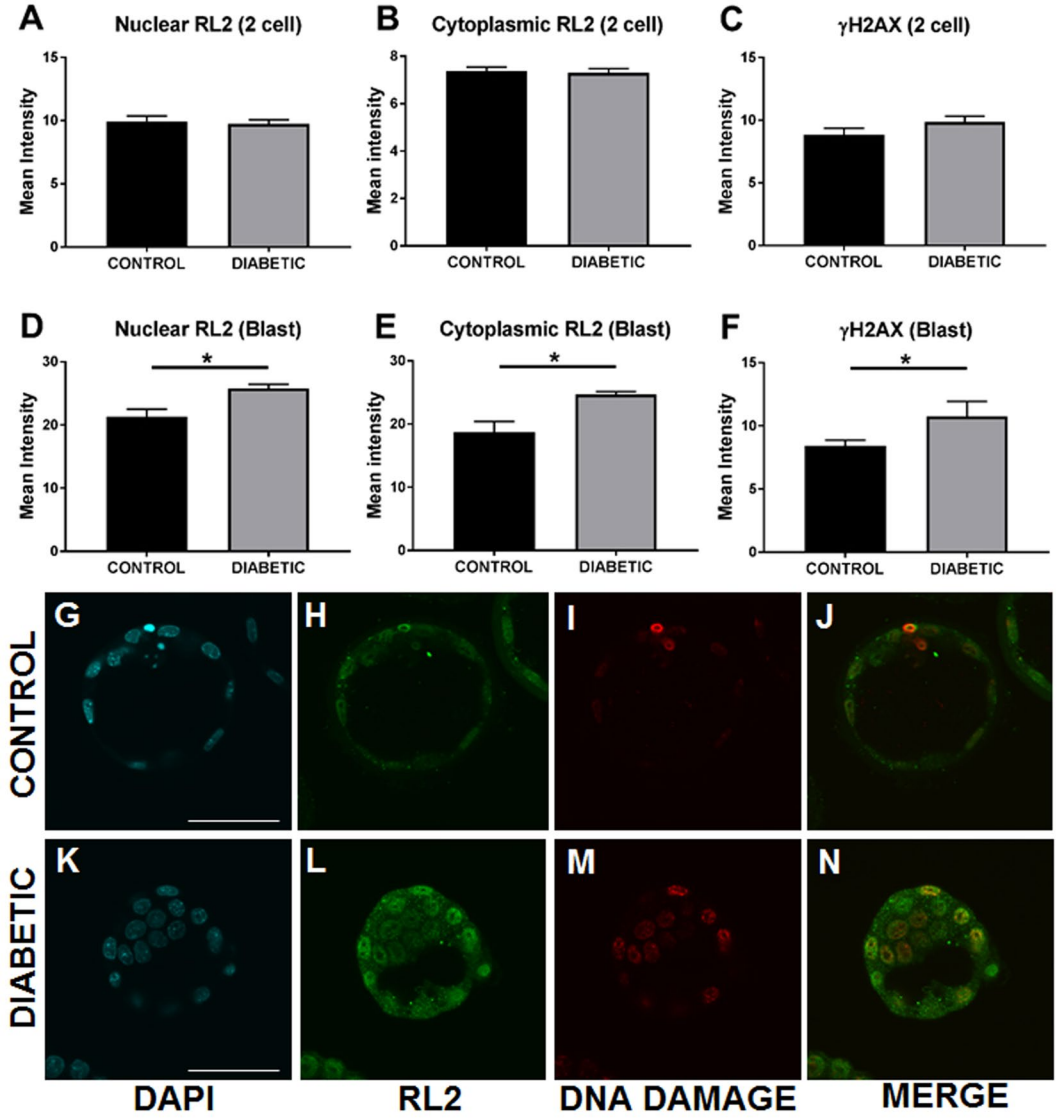
Pre-gestational diabetes increases *O*-GlcNAc and DNA damage in blastocyst-stage embryos. Control (black) and diabetic (grey) female mice were hyperstimulated and mated, then embryos were flushed from oviducts on d1.5 p.c (**A**–**C**) and uterus on d3.5 p.c (**D**–**N**). Nuclear and whole embryo *O*-GlcNAc (RL2 antibody, green **A**,**B**,**D**,**E**,**H**,**L**) was assessed by immunohistochemistry followed by image analysis. DNA damage (ɣH2AX, red, **C**,**F**,**I**,**M**) was assessed using immunohistochemistry followed by image analysis (3–4 independent experimental replicates). Representative images of blastocysts from control (**G**–**J**) and diabetic (**K**–**N**) are shown. Scale bar represents 50 μm. Embryos were counterstained with DAPI nuclear stain (**G**,**K**). Data is presented as mean + SEM and effects of treatment were analysed by unpaired Student’s t-test. *P < 0.05.

To examine the effect of streptozotocin-induced diabetes on early development, embryos were flushed from oviducts recovered on d1.5 and d3.5 p.c from diabetic female mice. There was no impact of periconception-onset diabetes on the incidence of conception, with all mated mice yielding 2-cell cleavage-stage embryos on d1.5 p.c. (Fig. 1A). The total number of embryos was unchanged (Fig. 1B), however the number of viable embryos was significantly decreased (Fig. 1C) after streptozotocin treatment. At d3.5 p.c., fewer viable embryos were flushed from the uterus (Fig. 1D,E) and many had evidence of arrest at cleavage or morula stages with a 60% reduction in their development to blastocyst (Fig. 1F). γH2AX was used to assess DNA damage in these embryos and RL2 was used to assess β-*O*-GlcNAc status. Two-cell embryos flushed from the oviduct displayed no change in either DNA damage or O-GlcNAc (Fig. 2A–C). In contrast, blastocysts flushed from tracts of diabetic females on d3.5 p.c. displayed increased levels of both nuclear and cytoplasmic *O*-GlcNAc (Fig. 2D–E,H,L) and nuclear DNA damage (Fig. 2F,I,M). Within blastocysts, there was clear co-localisation of DNA damage and elevated *O*-GlcNAc glycosylation in individual blastomeres (Fig. 2J,N).

### Periconception-onset diabetes alters mid-gestation conceptus development and fetal viability

**Figure 3:**
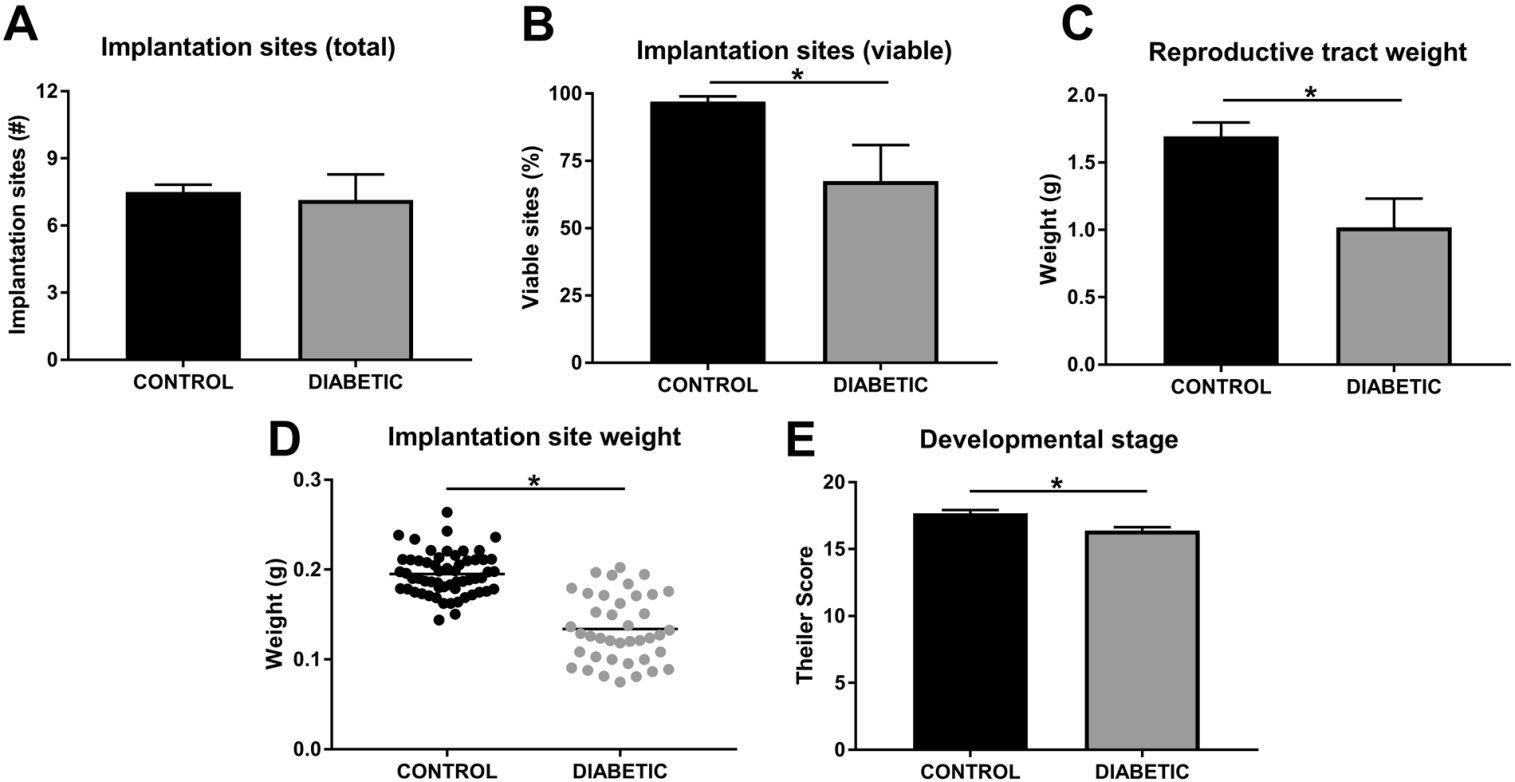
Periconception-onset diabetes causes midgestation fetal loss, impaired fetal growth and delayed fetal development. Control (black) and diabetic (grey) female mice were naturally mated and conceptus tissue was assessed on d11.5 p.c. Total implantation site number (**A**), percent viable implantation sites (**B**), reproductive tract weight (**C**), whole implantation site weight (**D**), and Theiler developmental score (**E**) were assessed (n = 15–16 mated females per treatment). Data is presented as mean (**D**) or mean + SEM and effects of treatment were analysed by unpaired Student’s t-test. *P < 0.05.

To examine the effect of streptozotocin-induced periconception-onset diabetes on mid-gestation development, pregnant diabetic female mice were autopsied on d11.5 p.c. There was no difference between treatment groups in the total number of implantation sites (viable and non-viable; Fig. 3A) however due to elevated rates of early fetal resorption the number of viable implantation sites was decreased (Fig. 3B), reflected in decreased reproductive tract weight (Fig. 3C). In viable implantation sites, there was a 30% decrease in the mass of individual conceptus units (fetus plus placenta) (Fig. 3D), and when fetal development was assessed according to Theiler score, a delay corresponding to approximately 24 hours was evident (Fig. 3E).

### Periconception-onset diabetes increases protein *O*-GlcNAcylation and activation of stress deflection pathways in reproductive tract tissues

**Figure 4:**
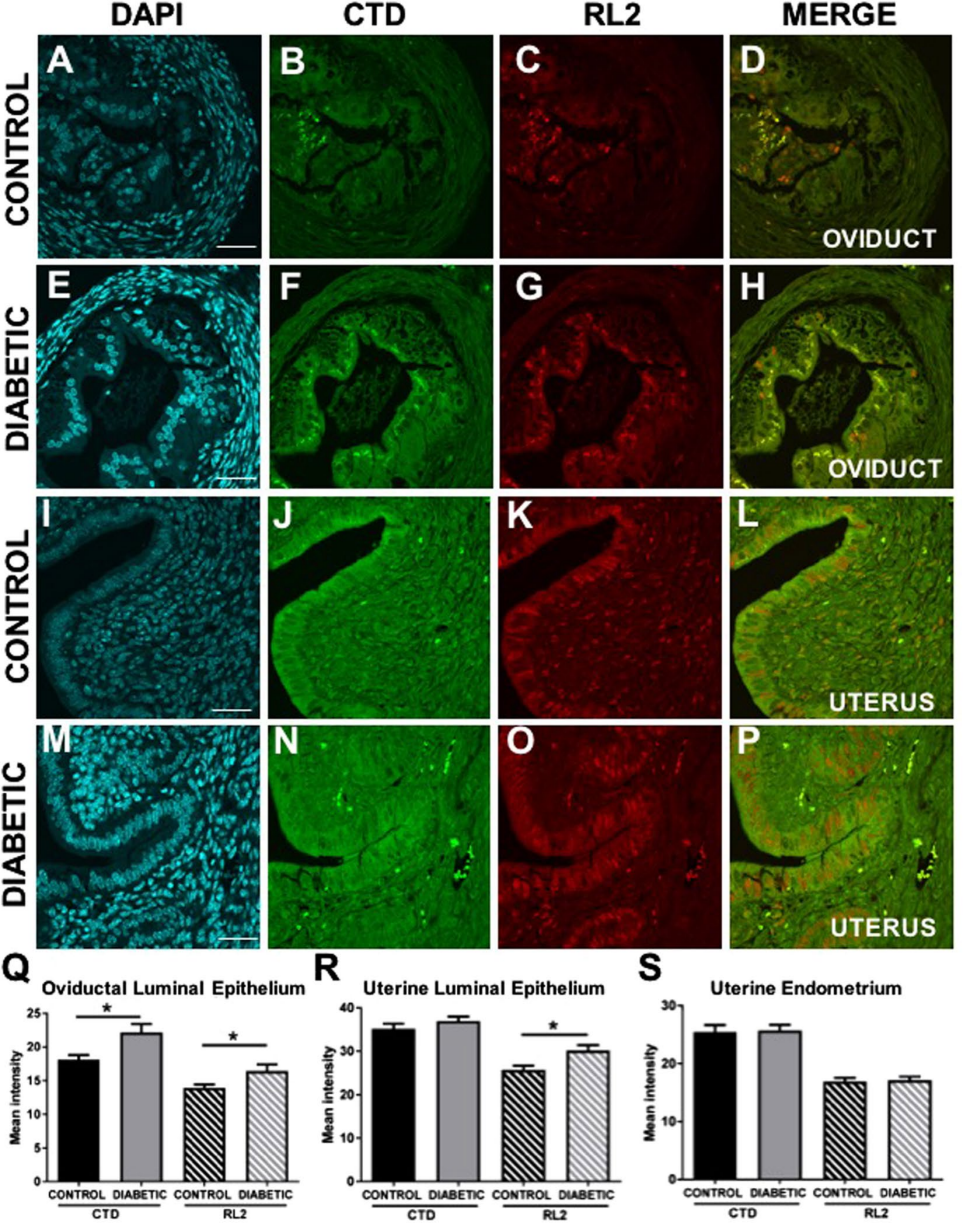
Periconception-onset diabetes increases *O*-GlcNAc in the female reproductive tract during the preimplantation period. Control (black) and diabetic (grey) female mice were hormone-stimulated and mated, then oviducts (day 1.5 pc, **A**–**H**,**Q**) and uterus (day 3.5 pc, **I**–**P**,**R**–**S**) were fixed for immunohistochemical analysis of *O*-GlcNAc (CTD antibody, green, closed bars; RL2 antibody, red, striped bars), and counterstained with DAPI (blue). Scale bar represents 50 μm. Representative images are shown of oviduct (**A**–**H**) and uterus (**I**–**M**) from control (**A**–**D**,**I**–**L**) and diabetic (**E**–**H**,**M**–**P**) mice. Images of oviductal (**Q**) and uterine (**R**) luminal epithelium and endometrium (**S**) were analysed using FIJI in 20 sections per mouse, from 6 mice per treatment group. Data is presented as mean + SEM staining intensity and effects of treatment were analysed by unpaired Student’s t-test. *P < 0.05.

To examine the effect of streptozotocin-induced periconception-onset diabetes on the abundance of *O*-GlcNAc in the oviduct (d1.5 p.c.) and uterus (d3.5 p.c.), two antibodies were used to detect *O*-GlcNAc (Fig. 4, green CTD, red RL2). Treatment significantly increased the abundance of *O*-GlcNAc in the luminal epithelium of the oviduct (Fig. 4A–H,Q), with a distinct pattern localising to the cytoplasmic region immediately subjacent to the luminal surface. Although weak staining was similarly localised in controls, the staining intensity was notably higher in treated mice. By d3.5 p.c., when the blastocyst is within the uterus, there was an increased abundance of *O*-GlcNAc in the uterine luminal epithelium of diabetic females (Fig. 4I–P,R), while no difference was evident in the endometrial stroma (Fig. 4P). To examine if the effect extended throughout the reproductive tract, we also examined the abundance of *O*-GlcNAc in the ovary in the streptozotocin-treated mice on d1.5 p.c. of pregnancy, but no difference in staining was evident (Suppl. Figure 1).

**Figure 5:**
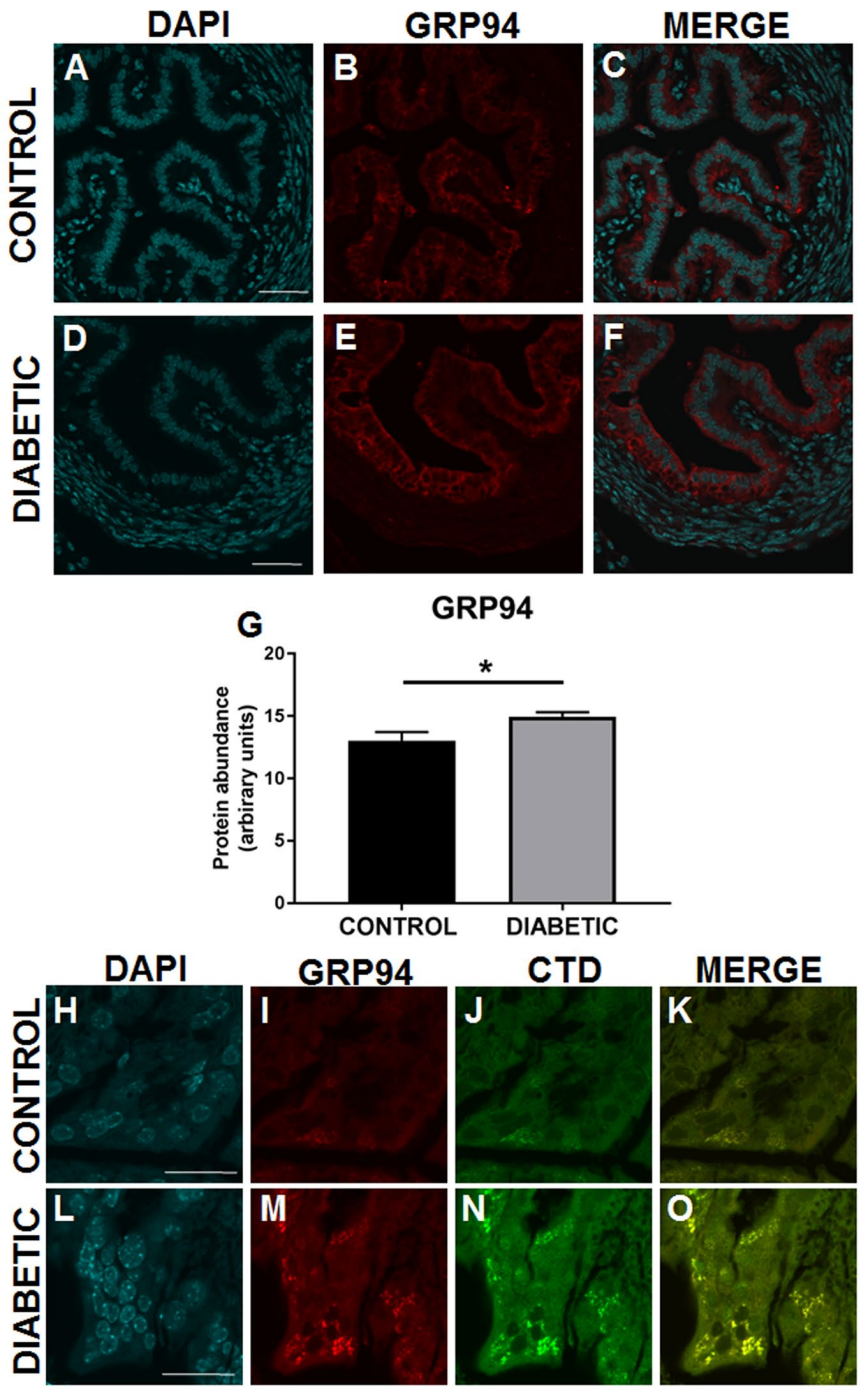
Periconception-onset diabetes is associated with elevated GRP94 abundance, and GRP94-*O*-GlcNAc co-localisation in the oviduct. Control (**A**–**C**, **H**–**K**, black) and diabetic (**D**–**F**,**L**–**O**, grey) female mice were hyperstimulated and mated, then oviducts were recovered on day 1.5 pc and fixed for immunohistochemical analysis of GRP94 (red, **A**–**F**,**I**,**M**) and *O*-GlcNAc (CTD antibody, green, **J**,**N**) and counterstained with DAPI (blue). Scale bar represents 50 μm. Image analysis (**G**) was performed on oviductal luminal epithelium using FIJI in 20 sections per mouse from 6 mice per treatment. Data is presented as mean + SEM staining intensity and effects of treatment were analysed by unpaired Student’s t-test. *P < 0.05.

We also examined the abundance of several heat shock protein family chaperones, which are known to be regulated by glucose (also known as the glucose regulated proteins; Grps) and which we have shown to be targets of *O*-GlcNAclyation in the cumulus-oocyte complex^15^. Endoplasmic-specific heat shock protein HSP90B1 (GRP94) was significantly increased in the oviductal epithelium on d1.5 p.c. particularly in the cytoplasmic region adjacent to the luminal surface (Fig. 5E). This was accompanied by loss of HSP90AA1 (Hsp90a) in the oviductal epithelium of diabetic females (Suppl. Figure 2E,M), and an increase in HSPA5 (GRP78), which is known to be induced by conditions which promote accumulation of unfolded proteins in the endoplasmic reticulum (ER) (Suppl. Figure 2G–L,N). The HSP family of chaperones are a well-characterised target of OGT^21,22^, and high levels of co-localisation of HSP90B1 and *O*-GlcNAc (Fig. 5H–O) were observed, suggesting HSP90 or other ER-localised proteins are targeted.

### Periconception-onset diabetes induces proinflammatory cytokine expression in the uterus and imbalance in the T cell response required for immune adaptation to pregnancy

**Figure 6:**
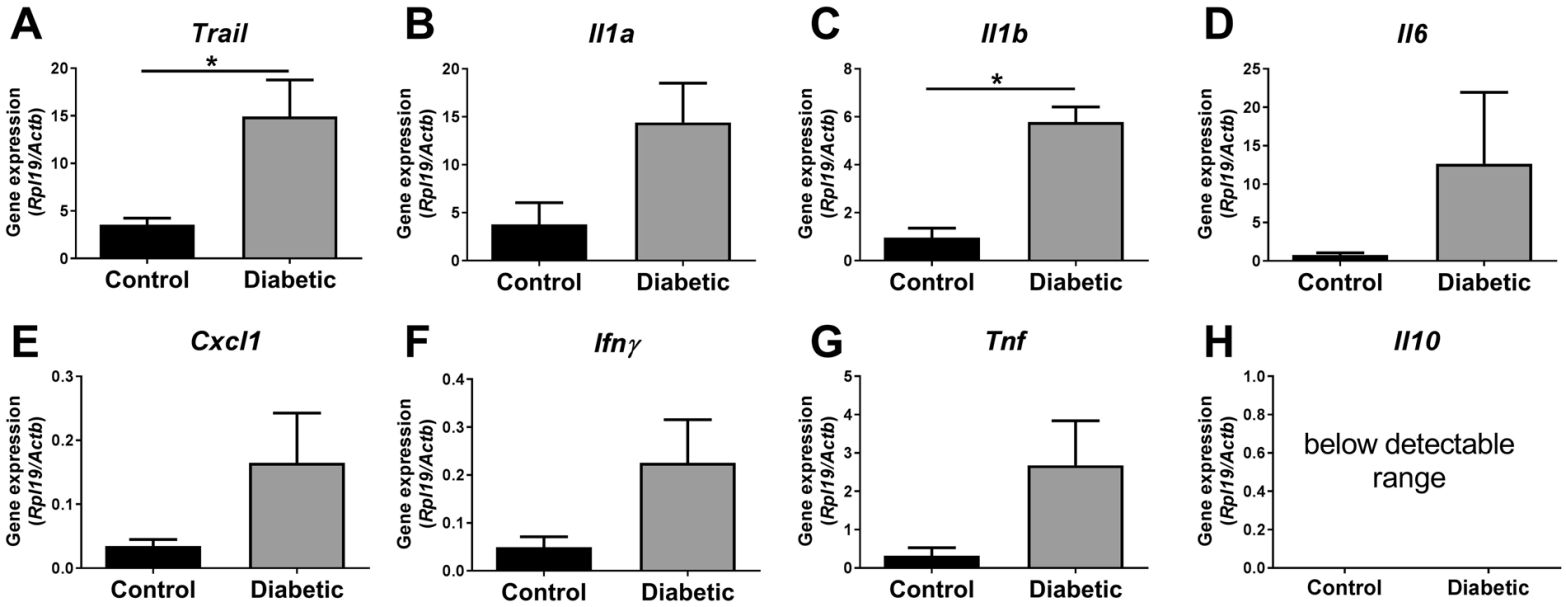
Periconception-onset diabetes is associated with pro-inflammatory cytokine expression in the uterus in early pregnancy. Control (black) and diabetic (grey) female mice were hormone-stimulated and mated, then the uterus was collected on d3.5 p.c. for cytokine analysis. mRNAs for proinflammatory cytokines (**A**) tumor necrosis factor-related apoptosis-inducing ligand (*Trail*), (**B**) interleukin 1a (*Il1a*), (**C**) interleukin 1b (*Il1b*), (**D)** interleukin 6 (*Il6*), (**E**) chemokine (C-X-C motif) ligand 1 (*Cxcl1*), (**F**) interferon gamma (*Ifng*) and (**G**) tumor necrosis factor (*Tnf*) and (**H**) anti-inflammatory cytokine interleukin *10* (*Il10*), were quantified by qPCR. Data is presented as mean + SEM from 5–7 mice per treatment group and effects of treatment were analysed by unpaired Student’s t-test. *P ≤ 0.05.

Given the biochemical and growth anomalies described above, and the known role of cytokines and T-cells for implantation success^23^, we characterised the impact of periconception-onset diabetes on the immune milieu of early pregnancy. In the uterus of diabetic females on the day of implantation (d3.5 p.c.), we demonstrated substantial upregulation of pro-inflammatory cytokines including *Trail*, *Il1b* (both P < 0.05), as well as trends towards elevated *Il1a*, *Ifng* and *Tnf* (all P < 0.07) (Fig. 6).

**Figure 7:**
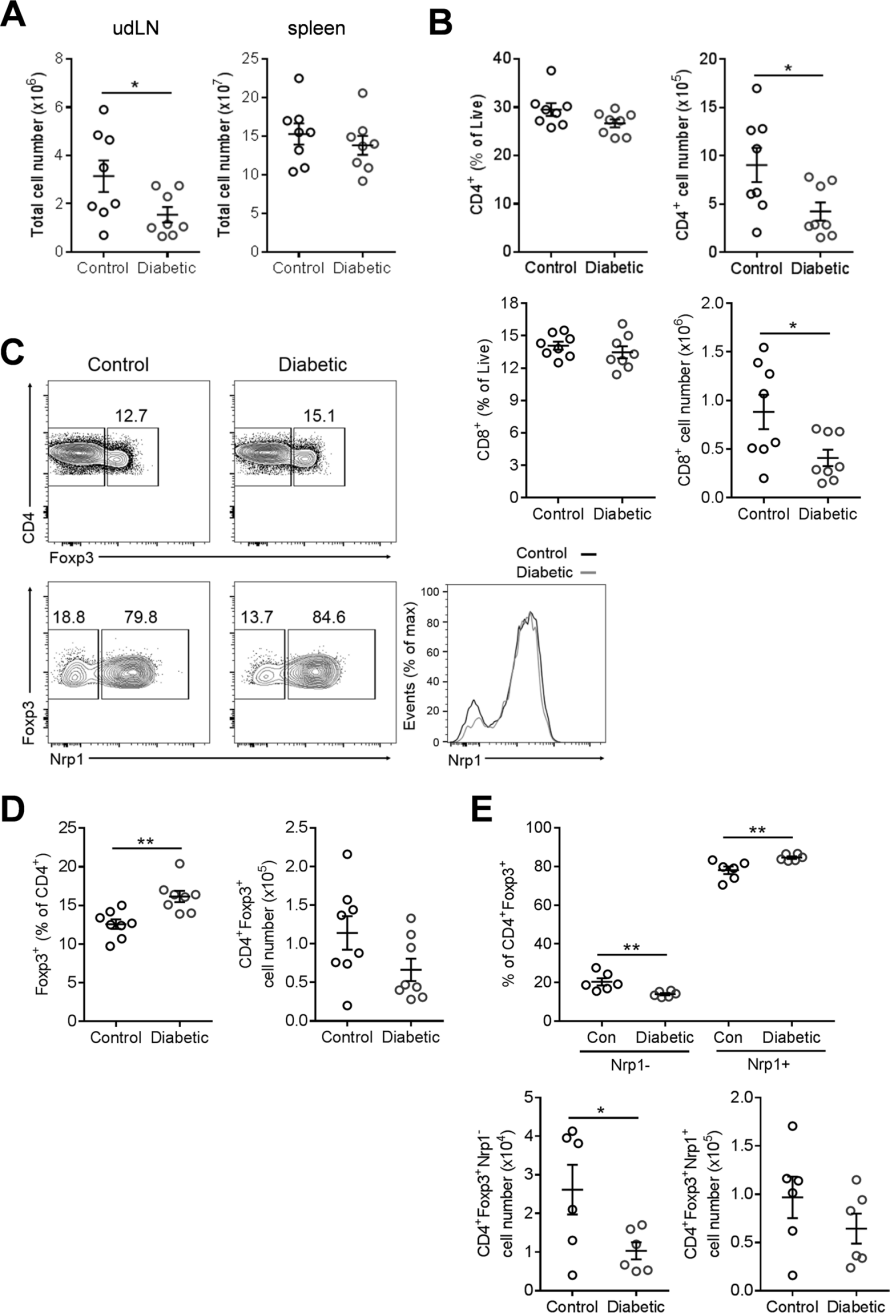
Periconception-onset diabetes diminishes total leukocytes and perturbs Foxp3+ Treg cell frequency in the uterus-draining PALN in early pregnancy. Control and diabetic female mice were hormone-stimulated and mated, then on day 3.5 of pregnancy, leukocyte subsets were quantified by flow cytometry. Total cell numbers in PALNs and spleens (**A**), and proportion and number of CD4^+^ and CD8^+^ T cells (**B**), Treg cells (CD4^+^Foxp3^+^) (**D**), peripherally-induced Treg cells (pTreg; CD4^+^Foxp3^+^Nrp1^−^) and thymus-derived Treg cells (tTreg; CD4^+^Foxp3^+^Nrp1^+^) (**E**) in PALNs are depicted. (**C**) Representative flow cytometric analysis of Foxp3 staining in CD4^+^ T cells and Nrp1 staining in CD4^+^Foxp3^+^ Treg cells, in PALNs, and representative histogram depicting mean fluorescence intensity (MFI) of Nrp1 in Treg cells. (**A**,**B**,**D**,**E**) n = 6–8 mice per treatment group. Data are presented as mean + SEM and effects of treatment were analysed by unpaired Student’s t-test (*p < 0.05, **p < 0.01).

T cell phenotypes and absolute numbers in the spleen and PALN, the latter a major site of maternal-fetal immune regulation and source of T cells recruited to the implantation site, were determined by flow cytometry immediately prior to embryo implantation on d3.5 p.c. We focused on T lymphocytes, particularly regulatory T (Treg) cells, as these are known to be key mediators of maternal immune tolerance required for implantation success^23^. Total T-cell numbers were decreased in the uterus-draining para-aortic lymph nodes (PALN) of diabetic females (P < 0.05) (Fig. 7A), as were total CD4^+^ and CD8^+^ T-cells (Fig. 7B). These changes were not evident in the spleen (Fig. 7A and Suppl. Figure 4), indicating an impact of diabetes specific to the immune response to conception, rather than a systemic effect. Despite preferential sparing of the CD4^+^Foxp3^+^ Treg cell population (%CD4^+^ T cells), there was a trend to reduced total CD4^+^ Foxp3^+^ Treg cells (P = 0.08) (Fig. 7D). Amongst the Treg cells peripherally-induced Treg (pTreg) cells and thymically-derived Treg (tTreg) cells can be distinguished by expression of neuropilin-1 (Nrp1) (Fig. 7C). Within the PALN but not the spleen there was a striking shift in the proportion of these two Treg cell subsets in diabetic females, such that mean absolute numbers of Nrp1^−^ pTreg cells were decreased by 60% (P < 0.01) while Nrp1^+^ tTreg cells were not significantly changed (Fig. 7E). This observation indicates substantial impairment of the adaptive immune response to pregnancy in streptozotocin-treated mice.

## Discussion

**Figure 8:**
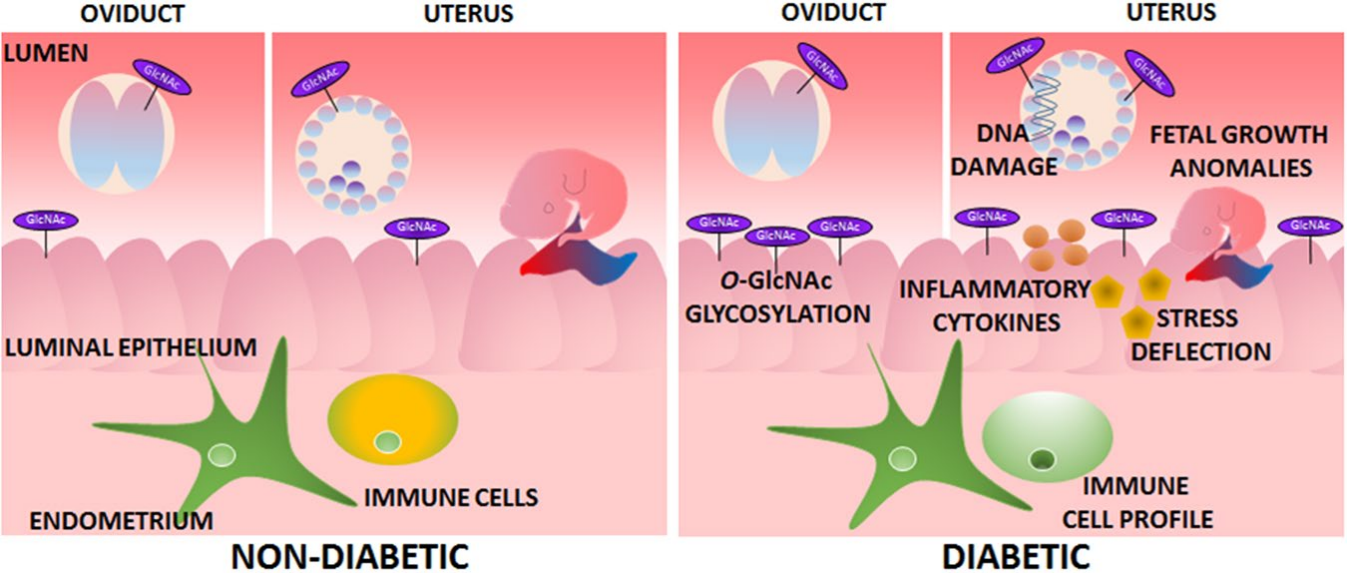
Periconception-onset diabetes alters the reproductive tract environment in early pregnancy. A schematic summary of the changes seen in early pregnancy in the streptozotocin-induced diabetic model.

The reproductive tract environment at conception profoundly influences the progression of pregnancy and the perinatal and long-term health of infants. During the first days of life, the embryo senses and adapts to the environment within the oviduct and uterus, setting in train a developmental program that will determine fetal growth and influence health trajectory after birth^24^. For the first time, we have described changes in *O*-GlcNAcylation of proteins in the early preimplantation embryo caused by streptozotocin-induced diabetes, and demonstrate that the reproductive tract is also prone to altered *O*-GlcNAc levels. We further demonstrate that this is associated with increases in ER-stress related response mechanisms, evidenced by altered levels of heat-shock pathway chaperones, and induces a pro-inflammatory environment accompanied by changes in the local cytokine and immune cell milieu that would impair maternal immune receptivity for implantation and pregnancy progression (Fig. 8).

In this study, we report evidence which supports that the embryo is responding to the external physiological cues of the diabetic state, as indicated by compromised blastocyst development and the increased levels of blastocyst protein *O*-GlcNAcylation. In other paradigms of hyperglycaemia and hyperinsulinaemia, an abnormal increase in *O*-GlcNAc has been observed, resulting in a disruption in the balance of protein modification processes involved in controlling cell functions^25^. Dysregulation of *O*-GlcNAcylation is now an established mechanism causing dysfunction of insulin signalling and glucose toxicity; significant characteristics of type 2 diabetes^25^. Additionally, alteration in *O*-GlcNAc protein modification is thought to underpin other diabetic-related pathologies, including cardiomyopathy, erectile dysfunction and neurodegenerative diseases, due to a systemic elevation in *O*-GlcNAcylation^26–28^. However, this has not previously been explored within the diabetic reproductive tract or in *in vivo*-derived embryos.

We observed elevated DNA damage in diabetic embryos, with distinct co-localisation between the DNA-damage histone marker γH2AX (phosphorylation at Serine 139), and *O*-GlcNAcylation. In response to DNA damage, multiple repair factors relocate to the sites of damage to activate repair and cell cycle checkpoints^29^. The dynamic relocation of DNA repair factors is mediated by DNA damage-induced post-translational modifications on histones and their binding partners at, or adjacent to, the sites of DNA damage^30^, one of which includes the phosphorylation of histone H2AX (aka γH2AX). A recent report describes *O-*GlcNAc transferase (OGT) locating to the site of DNA damage, with *O-*GlcNAc glycosylation of histone H2AX reducing the phosphorylation events, and helping cells recover from DNA damage^31^. In addition to the targeting of histone H2AX, there are many other nuclear targets for *O-*GlcNAcylation. Utilising precursors derived from metabolic flux, *O-*GlcNAc functions as a homeostatic regulator of nuclear behaviour, in a mechanism recently termed ‘metaboloepigenetics’^32^. Alterations to nutrients and environmental cues are tied to fluctuations in metabolic behaviour, in turn causing fluctuations in intermediate metabolites which serve as substrates or cofactors for epigenetic modification (reviewed in ref.^33^). Histones, the polycomb repressor complex and RNA polII are just a few of the characterised targets for nuclear *O-*GlcNAc glycosylation^34^ and provide plausible mechanism by which an epigenetic change may underpin the transmission of diabetes from mother to offspring *in utero*.

Several studies describe the impact of environmental perturbation in the conception phase on early embryo biochemistry and molecular biology^35^, but how these link to long-term programming of offspring is not clear. The immunological state within the reproductive tract shapes embryonic programming, in part through direct effects of cytokines on the embryo and in part through influencing maternal tract receptivity and placental morphogenesis^36^. Immune-modulating cytokines are pivotal for mediating communication between the maternal tract and the embryo. Compelling evidence shows that cytokines emanating from the oviduct and uterus can ‘fine-tune’ embryo development during the peri-conception period, influencing a range of cellular events from cell survival and metabolism, through division and differentiation, and potentially impart long-term impact through epigenetic remodeling^37^. The relative balance between survival agents and apoptosis-inducing agents influence the course of preimplantation development, causing embryos to adapt to varying maternal environments. In a healthy state, embryotrophic factors protect embryos from cell stress and support them to thrive^36^. In contrast, with excessive inflammation, embryotoxic cytokines such as TRAIL, TNF and IFNG elicit cell stress in embryos and depending on their levels, can cause embryo developmental arrest and demise^38^. Our observation of elevated expression of embryotoxic cytokines TRAIL, and a trend towards increased IL1A, TNF and IFNG in hyperglycemic conditions, provides a direct mechanism contributing to the impact of diabetes on altered developmental trajectory of offspring, and support earlier reports of elevated uterine TNF and reduced TGFB2 and LIF accompanied by impaired blastocyst development in diabetic rat and mouse models^39–41^.

In addition to the altered uterine cytokine profile, we found striking changes in the immune cells of the local lymph nodes in diabetic females at implantation. As the developing fetus expresses paternal transplantation antigens, is it susceptible to a potentially detrimental maternal immune response. In successful pregnancy, the effects of hormones and seminal fluid at coitus cause maternal T cells to proliferate in the PALN. In healthy conception, paternal antigen-specific CD4^+^Foxp3^+^ Treg cells accumulate in response to seminal fluid and cytokines, and are then recruited to the uterus to promote a tolerogenic environment for successful implantation and to support robust fetal and placental development^23,42,43^. Two main subsets of Treg cells exist, both with recognised roles in mediating maternal tolerance during pregnancy; tTreg cells, derived from the thymus, and pTreg cells, induced via the peripheral conversion of conventional CD4^+^ T cells^43–45^. pTreg cell induction during early pregnancy is particularly critical and their genetic ablation disrupts placental morphogenesis and causes fetal loss in mice^45^. In human, perturbations in Treg cells and tolerance induction are associated with fetal growth restriction, preeclampsia and preterm birth^46,47^.

We observed lymphopenia in the PALNs draining the uterus in diabetic females, with less than half the expected CD4^+^ and CD8^+^ T cell population at the time of embryo implantation, when T-cell proliferation to generate the facilitating maternal immune response to pregnancy is normally initiated^48^. Despite relative protection of Treg cells compared to the CD4^+^ effector cell compartment, consistent with previous reports in the streptozotocin-induced diabetes model^49^, potentially adverse changes within the Treg cell compartment were evident in diabetic females. Notably we observed diminished pTreg cell populations in the PALN at implantation. These changes in T cell dynamics presumably indicate impaired T cell activation and proliferation, although elevated apoptosis is also possible. It seems likely that these changes are secondary to the elevated pro-inflammatory cytokine expression observed in the reproductive tract since these cytokines impact the phenotype of antigen-presenting cells that determine the course of immune adaptation for pregnancy^50^. Since dendritic cells are exposed to uterine cytokines as they take up antigen and exit to draining lymph nodes, a shift towards an inflammatory phenotype in the reproductive tract would likely be sufficient to skew the immune environment in the draining LNs. Collectively, these data indicate that diabetes-induced hyperglygaemia causes an immune imbalance in the reproductive tract and PALNs and a suboptimal induction of pTreg cells at implantation. Since Treg cells exert effects on uterine vascular adaptation and placental development^45^, a change in their abundance and phenotype at implantation can be reflected in altered fetal growth and survival in later gestation. Other studies show clearly that skewed phenotypes in the maternal T cell response secondary to an altered cytokine environment at conception can constrain fetal development in mid-gestation^51^. The pro-inflammatory immune environment induced by peri-conception diabetes thus provides a plausible biological mechanism, for programming the altered fetal growth seen in this model.

The mechanism by which diabetes induces inflammatory cytokines in the female reproductive tract is not clear, but this is a well-known consequence of diabetes-induced hyperglycaemia in other tissues (reviewed in refs^52,53^). One biologically plausible mechanism is that elevated stress response in oviductal and uterine epithelial cells secondary to hyperglycaemia promotes their release of TRAIL and other pro-inflammatory cytokines. Based on previous studies^54–56^ it seems likely that hyperglycaemic is the cause of the inflammatory response. However a limitation of the streptozotocin model is that direct toxic effects of the streptozotocin cannot be excluded, although this seems highly unlikely given that the half-life of this chemical is 15–30 minutes^57^ and cytokine expression was quantified at least 9–12 days after streptozotocin administration. Consistent with a role for the stress response, we found distinct changes in the abundance and localisation of stress deflection proteins GRP94 (HSP90B1), HSP90AA1 and HSPA5 in the luminal epithelium of the oviduct in early pregnancy. The pattern of regulation described is consistent with activation of ER stress and the unfolded protein response (UPR), reported in diabetes previously^58^. Of particular interest is the downregulation of HSP90α, which is known to be important for processing of antigens in the ER in preparation for presentation via MHC Class I^59^. Given the importance for appropriate paternal antigen presentation for the activation of a tolerant maternal immune response in early pregnancy^60^, we speculate that the shift in the HSP chaperone proteins may disrupt the immune cell cross-talk required to establish a healthy pregnancy. Excitingly, pharmacological interventions to suppress stress deflecting pathways have been demonstrated to be effective in pregnancy^61^, so may form future therapeutic targets for consideration in maternal diabetes.

In conclusion, we have identified that the pre-implantation embryo and reproductive tract are dramatically altered by streptozotocin-induced periconception-onset diabetes. We report that the embryo is susceptible to hyper-*O*-GlcNAcylation of both cytoplasmic and nuclear proteins, and this accompanies DNA damage. We show changes to cellular function of the oviductal and uterine epithelium during early pregnancy, including disruption of the heat shock pathway and upregulation of a number of pro-inflammatory cytokines that amplify cell stress in embryos and cause changes to the local immune environment. Each of these pathophysiological changes presumably synergise to impact the events of embryo implantation and robust placentation required for optimal fetal growth. This work identifies molecular and physiological disruptions to the embryo, female reproductive tract and immune system in early pregnancy, all of which we speculate could also occur in diabetic women. If aspects of this pathophysiological response are recapitulated in women, this would imply that reduced fertility in diabetic women may be secondary to a shift in the peri-conception stress response, and cytokine and immune environment. If indeed evidence for a periconception impact of maternal diabetes continues to build, preconception screening and treatment interventions will likely have a greater clinical impact than interventions in later pregnancy.

## Materials and Methods

### Animal handling and models

Mice were purchased from Animal Resources Centre, Perth, maintained in 14 h/ 10 h light dark conditions and given water and rodent chow *ad libitum*. All experiments were approved by The University of Adelaide Animal Ethics Committee (M-2013–233/172) and conducted in accordance with the Australian Code of Practice for the Care and Use of Animals for Scientific Purposes. Eight week old female C57BL/6 mice were treated with 180 mg/kg streptozotocin dissolved in citrate buffer (pH 4.5). Blood glucose was monitored every two days by tail prick (Accu-Chek, Roche, Sydney, NSW, Australia). When blood glucose reached 14 mmol/L (6–9 days following injection), mice were available for experiments. For analysis on day (d) 1.3 post-coitus (p.c.) and d3.5 p.c., female mice were administered (i.p.) 5 IU equine chorionic gonadotrophin (eCG; Folligon, Intervet, Boxmeer, The Netherlands). Forty-six hours post-eCG injection, mice were administered 5 IU human chorionic gonadotrophin (hCG; hCG/Pregnyl; Merck, Kilsyth, VIC, Australia) and mated with males of the same strain. The following morning, considered d0.5 p.c., mice were assessed to confirm the presence of copulation plugs and then culled via cervical dislocation on d1.5 or d3.5 p.c. For mid-gestation pregnancy parameters, female mice were placed with males without prior hormone treatment and assessed daily for copulation plugs then killed and autopsied on d11.5 p.c.

### Tissue and embryo collection

Unless otherwise specified, all reagents and antibodies were purchased from Sigma-Aldrich (St Louis, MO, USA). Embryos and reproductive tracts were collected into Research Vitro Wash (Cook Medical Pty. Ltd.) on warming stages calibrated to 37 °C. On d1.5 and 3.5 p.c., embryos were flushed into media followed by fixation (30 min, 4% paraformaldehyde in phosphate-buffered saline (PBS) + 3 mg/ml polyvinylalcohol). Oviducts/uteri were snap frozen in liquid nitrogen for qPCR analysis or fixed O/N (as above) for histological analysis. On d3.5 p.c., spleen and uterus-draining para-aortic lymph nodes (PALN) from females confirmed pregnant by embryo flushing, were harvested for flow cytometry analysis. On d11.5 p.c., reproductive tracts and implantation sites were dissected and weighed, and embryos staged using the Theiler system.

### Immunohistochemistry, confocal microscopy and image analysis

Embryos: Following fixation, embryos were analysed by immunohistochemistry for DNA damage repair (anti-γH2AX primary antibody; Cell Signaling Technology, Danvers, MA, USA) and *O*-GlcNAc (pan O-GlcNAc RL2 antibody; Abcam, Melbourne, VIC, Australia) in three experimental replicates, with at least 15 embryos per replicate using published methods^15^. *Ovary*, *oviduct and uterus:* 7 µm paraffin embedded tissue sections were analysed for glycosylation and stress deflection (RL2 and HSP90a: Abcam, Melbourne, VIC, Australia, Pan *O*-GlcNAc CTD110.6: (Sigma), GRP94 and HSPA5: Novusbio, Littleton, CO, USA) in six mice/treatment/time point (20 sections/mouse) using standard methods^62^. Fluorescence was detected using a Fluoview FV10i Confocal Laser-Scanning Microscope (Olympus) and instrument settings were constant for each replicate. Images obtained were processed and analyzed using Fiji software (National Institutes of Health), with pre-developed plugins and macros. For embryos, regions of interest were whole cells and nuclei only, and cytoplasmic staining was the difference between these values; the blastocoel cavity was excluded from blastocyst analysis. For reproductive tract tissues, regions of interest were the oviductal epithelium and uterine epithelium (details; figure legends).

### RNA isolation, cDNA synthesis and qPCR

Total RNA was isolated using the RNeasy Mini Kit (Qiagen, Chadstone Centre, VIC, Australia), per the manufacturer’s instructions. First-strand cDNA was synthesized from total RNA using random hexamer primers and First Strand cDNA Synthesis Kit (Life, Invitrogen, Australia Pty. Ltd.). Gene primers for real-time qPCR (Supplementary Table 1) were designed from published mRNA sequences from the National Center for Biotechnology Information PubMed database using Primer Express software (Applied Biosystems, Foster City, CA) and synthesized by Geneworks (Hindmarsh, SA, Australia). qPCR was performed in triplicate for each sample on a Rotor-Gene 6000 (Corbett Life Science, Sydney, NSW, Australia) using standard methods described previously^62^. All gene expression was normalised to *Rpl19* and *Actb* amplified in parallel. Results were expressed as 2^−^(ΔCT).

### Flow cytometry

Cell suspensions were generated from spleens and PALNs by gentle homogenization in RPMI 1640 (Gibco^TM^, Life Technologies, Invitrogen, Australia Pty. Ltd.). Erythrocytes were lysed in splenocyte suspensions using red blood cell lysis buffer (155 mM NH_4_Cl, 10 mM KHCO_3_ and 0.1 mM EDTA). Dead cell exclusion was enabled by addition of BD Horizon™ Fixable Viability Stain 620 (Becton Dickinson (BD), Franklin Lakes, NJ). 10^6^ cells were stained in PBS (0.05% sodium azide, 0.1% BSA (Sigma). Fc receptors were blocked with anti-CD16/CD32 Fc Block™ (Mouse Fc Block™; BD) before surface staining with antibodies; anti-CD4 APC-Cy7 (GK1.5, BD), anti-CD8α PE-Cy7 (53–6.7, BD) and Nrp1 BV421 (3E12, Biolegend, San Diego, CA). Intracellular staining was performed using Foxp3 Staining Buffer Set (eBioscience, San Diego, CA), as per the manufacturer’s instructions, and anti-Foxp3 APC (FJK-16s, eBioscience) antibody. Data were acquired on a BD FACSCantoII. All data were analysed using FlowJo software (TreeStar, Inc., Ashland, OR).

### Statistical Analysis

Data are presented as mean ± SEM, and effects of treatment were analysed by one-way Student’s T Test using GraphPad Prism Version 7 and SPSS (Version 22) for Windows as described in the figure legends. Implantation size weight was expressed as estimated marginal means and analysed by mixed model linear repeated-measures analysis of variance and post-hoc least significant difference test, with mother as subject. PCR data were natural log-transformed where necessary to achieve equal variances before analysis. Embryo rate (%) data was arcsine transformed for analysis. Statistically significant difference was concluded when P < 0.05. All data is generated from >3 experimental replicates, unless stated otherwise.

### Data Availability

All data generated or analysed during this study are included in this published article (and its Supplementary Information files).

## Electronic supplementary material

Supplementary Info

## References

[1] Garcia-Vargas, L., Addison, S. S., Nistala, R., Kurukulasuriya, D. & Sowers, J. R. Gestational Diabetes and the Offspring: Implications in the Development of the Cardiorenal Metabolic Syndrome in Offspring. *Cardiorenal Med* **2**, 134–142, doi:000337734 (2012). 10.1159/000337734 PMC3376343 22851962

[2] Negrato CA, Mattar R, Gomes MB (2012). Adverse pregnancy outcomes in women with diabetes. Diabetol Metab Syndr.

[3] Temple RC, Aldridge VJ, Murphy HR (2006). Prepregnancy Care and Pregnancy Outcomes in Women With Type 1 Diabetes. Diabetes Care.

[4] Dunne FP (1999). Pregnancy outcome in women with insulin-dependent diabetes mellitus complicated by nephropathy. QJM.

[5] Ray JG, O’Brien TE, Chan WS (2001). Preconception care and the risk of congenital anomalies in the offspring of women with diabetes mellitus: a meta-analysis. QJM.

[6] Pearson DW, Kernaghan D, Lee R, Penney GC (2007). Scottish Diabetes in Pregnancy Study, G. The relationship between pre-pregnancy care and early pregnancy loss, major congenital anomaly or perinatal death in type I diabetes mellitus. BJOG.

[7] Casson IF (1997). Outcomes of pregnancy in insulin dependent diabetic women: results of a five year population cohort study. BMJ.

[8] El-Sayed YY, Lyell DJ (2001). New therapies for the pregnant patient with diabetes. Diabetes Technol Ther.

[9] Wyman A, Pinto AB, Sheridan R, Moley KH (2008). One-cell zygote transfer from diabetic to nondiabetic mouse results in congenital malformations and growth retardation in offspring. Endocrinology.

[10] Jawerbaum A, White V (2010). Animal models in diabetes and pregnancy. Endocr Rev.

[11] Olivier-Van Stichelen S, Wang P, Comly M, Love DC, Hanover JA (2017). Nutrient-driven O-GlcNAc cycling impacts neurodevelopmental timing and metabolism. J Biol Chem.

[12] Peterson SB, Hart GW (2016). New insights: A role for O-GlcNAcylation in diabetic complications. Crit Rev Biochem Mol Biol.

[13] Pantaleon M, Tan HY, Kafer GR, Kaye PL (2010). Toxic effects of hyperglycemia are mediated by the hexosamine signaling pathway and o-linked glycosylation in early mouse embryos. Biology of reproduction.

[14] Sutton-McDowall ML (2006). Glucosamine supplementation during *in vitro* maturation inhibits subsequent embryo development: possible role of the hexosamine pathway as a regulator of developmental competence. Biol Reprod.

[15] Frank LA (2014). Hyperglycaemic conditions perturb mouse oocyte *in vitro* developmental competence via beta-O-linked glycosylation of heat shock protein 90. Hum Reprod.

[16] Frank LA, Sutton-McDowall ML, Gilchrist RB, Thompson JG (2014). The effect of peri-conception hyperglycaemia and the involvement of the hexosamine biosynthesis pathway in mediating oocyte and embryo developmental competence. Mol Reprod Dev.

[17] Sutton-McDowall ML, Gilchrist RB, Thompson JG (2010). The pivotal role of glucose metabolism in determining oocyte developmental competence. Reproduction.

[18] Marth JD, Grewal PK (2008). Mammalian glycosylation in immunity. Nat Rev Immunol.

[19] Esposito K (2002). Inflammatory cytokine concentrations are acutely increased by hyperglycemia in humans: role of oxidative stress. Circulation.

[20] Robertson SA, Moldenhauer LM (2014). Immunological determinants of implantation success. Int J Dev Biol.

[21] Mollapour M, Neckers L (2012). Post-translational modifications of Hsp90 and their contributions to chaperone regulation. Biochim Biophys Acta.

[22] Overath T (2012). Mapping of O-GlcNAc sites of 20 S proteasome subunits and Hsp90 by a novel biotin-cystamine tag. Mol Cell Proteomics.

[23] Aluvihare VR, Kallikourdis M, Betz AG (2004). Regulatory T cells mediate maternal tolerance to the fetus. Nat Immunol.

[24] Hochberg Z (2011). Child health, developmental plasticity, and epigenetic programming. Endocrine reviews.

[25] Hart GW, Housley MP, Slawson C (2007). Cycling of O-linked beta-N-acetylglucosamine on nucleocytoplasmic proteins. Nature.

[26] Hurt KJ (2002). Akt-dependent phosphorylation of endothelial nitric-oxide synthase mediates penile erection. Proc Natl Acad Sci USA.

[27] Erickson JR (2013). Diabetic hyperglycaemia activates CaMKII and arrhythmias by O-linked glycosylation. Nature.

[28] Wells L, Whelan SA, Hart GW (2003). O-GlcNAc: a regulatory post-translational modification. Biochem Biophys Res Commun.

[29] Fernandez-Capetillo O, Celeste A, Nussenzweig A (2003). Focusing on foci: H2AX and the recruitment of DNA-damage response factors. Cell Cycle.

[30] Ismail IH, Hendzel MJ (2008). The gamma-H2A.X: is it just a surrogate marker of double-strand breaks or much more?. Environ Mol Mutagen.

[31] Chen Q, Yu X (2016). OGT restrains the expansion of DNA damage signaling. Nucleic Acids Res.

[32] Donohoe DR, Bultman SJ (2012). Metaboloepigenetics: interrelationships between energy metabolism and epigenetic control of gene expression. J Cell Physiol.

[33] Lewis BA (2014). & Hanover, J. A. O-GlcNAc and the epigenetic regulation of gene expression. J Biol Chem.

[34] Comer FI, Hart GW (2000). O-Glycosylation of nuclear and cytosolic proteins. Dynamic interplay between O-GlcNAc and O-phosphate. J Biol Chem.

[35] Eriksson UJ, Wentzel P (2016). The status of diabetic embryopathy. Ups J Med Sci.

[36] Robertson SA, Chin PY, Glynn DJ, Thompson JG (2011). Peri-conceptual cytokines–setting the trajectory for embryo implantation, pregnancy and beyond. Am J Reprod Immunol.

[37] Robertson SA, Chin PY, Schjenken JE, Thompson JG (2015). Female tract cytokines and developmental programming in embryos. Adv Exp Med Biol.

[38] Robertson, S. A., Chin, P. Y., Femia, J. G., Brown, H. M. Embryotoxic cytokines-Potential roles in embryo loss and fetal programming. *J Reprod Immunol.* **125, **80-88, doi: 10.1016/j.jri.2017.12.003 (2017) 10.1016/j.jri.2017.12.003 29306096

[39] Pampfer S (1995). Possible role for TNF-alpha in early embryopathy associated with maternal diabetes in the rat. Diabetes.

[40] Fein A (2002). Diabetes teratogenicity in mice is accompanied with distorted expression of TGF-beta2 in the uterus. Teratog Carcinog Mutagen.

[41] Roca V (2009). Potential immunomodulatory role of VIP in the implantation sites of prediabetic nonobese diabetic mice. Reproduction.

[42] Robertson SA (2009). Seminal fluid drives expansion of the CD4+CD25+T regulatory cell pool and induces tolerance to paternal alloantigens in mice. Biol Reprod.

[43] Rowe JH, Ertelt JM, Xin L, Way SS (2012). Pregnancy imprints regulatory memory that sustains anergy to fetal antigen. Nature.

[44] Guerin LR, Prins JR, Robertson SA (2009). Regulatory T-cells and immune tolerance in pregnancy: a new target for infertility treatment?. Hum Reprod Update.

[45] Samstein RM, Josefowicz SZ, Arvey A, Treuting PM, Rudensky AY (2012). Extrathymic generation of regulatory T cells in placental mammals mitigates maternal-fetal conflict. Cell.

[46] Sharma S (2014). Natural killer cells and regulatory T cells in early pregnancy loss. Int J Dev Biol.

[47] Peltier MR (2003). Immunology of term and preterm labor. Reprod Biol Endocrinol.

[48] Johansson M, Bromfield JJ, Jasper MJ, Robertson SA (2004). Semen activates the female immune response during early pregnancy in mice. Immunology.

[49] Muller YD (2011). Immunosuppressive effects of streptozotocin-induced diabetes result in absolute lymphopenia and a relative increase of T regulatory cells. Diabetes.

[50] Moldenhauer LM, Keenihan SN, Hayball JD, Robertson SA (2010). GM-CSF is an essential regulator of T cell activation competence in uterine dendritic cells during early pregnancy in mice. J Immunol.

[51] Moldenhauer LM, Diener KR, Hayball JD, Robertson SA (2017). An immunogenic phenotype in paternal antigen-specific CD8+T cells at embryo implantation elicits later fetal loss in mice. Immunol Cell Biol.

[52] Hu H (2015). AGEs and chronic subclinical inflammation in diabetes: disorders of immune system. Diabetes Metab Res Rev.

[53] Prattichizzo F (2017). Inflammageing and metaflammation: The yin and yang of type 2 diabetes. Ageing Res Rev.

[54] Ge H (2015). Functional relevance of protein glycosylation to the pro-inflammatory effects of extracellular matrix metalloproteinase inducer (EMMPRIN) on monocytes/macrophages. PLoS One.

[55] Gornik O, Lauc G (2008). Glycosylation of serum proteins in inflammatory diseases. Dis Markers.

[56] Goto Y, Uematsu S, Kiyono H (2016). Epithelial glycosylation in gut homeostasis and inflammation. Nat Immunol.

[57] Rossini AA, Like AA, Chick WL, Appel MC, Cahill GF (1977). Studies of streptozotocin-induced insulitis and diabetes. Proc Natl Acad Sci USA.

[58] Iwawaki T, Oikawa D (2013). The role of the unfolded protein response in diabetes mellitus. Semin Immunopathol.

[59] Moldenhauer LM, Hayball JD, Robertson SA (2010). Utilising T cell receptor transgenic mice to define mechanisms of maternal T cell tolerance in pregnancy. J Reprod Immunol.

[60] Kunisawa J, Shastri N (2006). Hsp90alpha chaperones large C-terminally extended proteolytic intermediates in the MHC class I antigen processing pathway. Immunity.

[61] Wu LL (2015). Mitochondrial dysfunction in oocytes of obese mothers: transmission to offspring and reversal by pharmacological endoplasmic reticulum stress inhibitors. Development.

[62] Brown HM (2015). Hemoglobin: a gas transport molecule that is hormonally regulated in the ovarian follicle in mice and humans. Biol Reprod.

