# Supplementary material for: Periconception onset diabetes is associated with embryopathy and fetal growth retardation, reproductive tract hyperglycosylation and impaired immune adaptation to pregnancy
Source: Sci Rep. 2018 Feb 1;8:2114. doi: 10.1038/s41598-018-19263-8 (PMC5794861; doi:10.1038/s41598-018-19263-8)
Supplement: Supplementary file 1 — Supplementary Info [file 41598_2018_19263_MOESM1_ESM.doc]

SUPPLEMENTARY INFORMATION

**Periconception onset diabetes is associated with embryopathy and fetal growth retardation, reproductive tract hyperglycosylation and impaired immune adaptation to pregnancy.**

*Short Title: Mechanisms of reproductive dysfunction in periconception-onset diabetes*

Hannah M Brown^1,2^ Ella S Green^1^, Tiffany CY Tan^1^, Macarena B Gonzalez^1^, Alice R Rumbold^1^, Louise Hull^1,3^, Robert J Norman^1,3^, Nicolle H Packer^4^, Sarah A Robertson^1*^ & Jeremy G Thompson^1,2*^.

*Authors contributed equally

1. Robinson Research Institute, Adelaide Medical School, University of Adelaide, Australia
2. Australian Research Council (ARC) Centre for Nanoscale Biophotonics, University of Adelaide, Australia
3. Fertility SA, Adelaide, Australia.
4. ARC Centre for Nanoscale Biophotonics, Department of Chemistry and Biomolecular Sciences, Macquarie University, Australia

**Corresponding author:**

Dr Hannah Brown

Robinson Research Institute, Centre for Nanoscale Biophotonics, Adelaide Medical School,

University of Adelaide, Level 3, Medical School South, Adelaide, Australia 5005

Email: [Hannah.brown@adelaide.edu.au](mailto:Hannah.brown@adelaide.edu.au)

Phone: +61 8 8313 8183

**SUPPLEMENTARY INFORMATION**

Table 1: Primer sequences for Real Time RTPCR.

| **Gene** | **Forward 5’-3’** | **Reverse 5’-3’** | **Gene Accession** |
| --- | --- | --- | --- |
| *Rpl19* | TTCCCGAGTACAGCACCTTTGAC | CACGGCTTTGGCTTCATTTTAAC | NM_026490 |
| *Actb* | TGTGATGGTGGGTATGGGTC | ACACGCAGCTCATTGTA | NM_007393 |
| *IL1a* | CCGACCTCATTTTCTTCTGG | GTGCACCCGACTTTGTTCTT | NM_010554.4 |
| *IL1b* | CCCAAGCAATACCCAAAGAA | GCTTGTGCTCTGCTTGTGAG | NM_008361.3 |
| *IL6* | ACAACCACGGCCTTCCCTAC | TCCACGATTTCCCAGAGAACA | NM_031168 |
| *Tnf* | GTAGCCCACGTCGTAGCAAAC | CTGGCACCACTAGTTGGTTGTC | NM_013693 |
| *Cxcl1* | ATTGTATGGTCAACACGCACG | TTTGAACGTCTCTGTCCCGAG | NM_008176 |
| *Trail* | CCAGAGATGCCGAGTACGGA | AAGGCTCCAAAGAAGCTGGCT | NM_009425 |
| *Ifng* | GCGTCATTGAATCACACCTG | TGAGCTCATTGAATGCTTGG | NM_008337.3 |
| *Il10* | AGGCGCTGTCATCGATTTCT | TGGCCTTGTAGACACCTTGGT | NM 010548.2 |

**SUPPLEMENTAL FIGURES**

**Figure 1: Pre-gestational diabetes has no effect on *O*-GlcNAc in the early pregnant corpus luteum.** Control and diabetic female mice were hormone-stimulated and mated, then ovaries were recovered on d1.5 p.c and fixed for immunohistochemical analysis of *O*-GlcNAc (CTD antibody, green, closed bars; RL2 antibody, red, hashed bars), and counterstained with DAPI (blue). Images represent the DAPI (A, E), CTD glycosylation (B, F), RL2 glycosylation (C, G). Image analysis was performed on ovaries using FIJI in 20 sections per mouse from 6 mice per treatment. Data is presented as mean + SEM staining intensity and effects of treatment were analysed by unpaired Student’s t-test. *P <0.05.

**Figure 2: Pre-gestational diabetes decreases HSP90a abundance, and increases HASA5 abundance in the oviduct.** Control (A-C, G-I, black) and diabetic (D-F, J-L, red) female mice were hormone-stimulated and mated, then oviducts were recovered on d1.5 p.c and fixed for immunohistochemical analysis of HSP90a (red, A-F, M) and HSPA5 (red, G-L, N) and counterstained with DAPI (blue). Image analysis (M-N) was performed on oviductal luminal epithelium using FIJI in 20 sections per mouse from 6 mice per treatment. Data is presented as mean + SEM staining intensity and effects of treatment were analysed by unpaired Student’s t-test. *P <0.05.

**Figure 3: Pre-gestational diabetes decreases lymph node hypertrophy in mid-gestation pregnant mice.** Control (top) and diabetic (bottom) female mice were naturally mated and then conceptus tissue was assessed at autopsy on d11.5 p.c. Images show size of uterine-draining (para-aortic) lymph nodes (white box). Lymph nodes appeared smaller in pregnant diabetic females.

**Figure 4: The effect of pre-gestational diabetes in early pregnancy on T lymphocytes in the spleen.** Control (black) and diabetic (red) female mice were hormone-stimulated and mated, then on day 3.5 of pregnancy, leukocyte subsets in the spleen were quantified by flow cytometry. Proportion and number of splenic CD4^+^ and CD8^+^ T cells (A), Treg cells (CD4^+^Foxp3^+^) (B), and proportion of peripherally-induced Treg cells (pTreg; CD4^+^Foxp3^+^Nrp1^-^) and thymus-derived Treg cells (tTreg; CD4^+^Foxp3^+^Nrp1^+^) (C) are depicted. (A, B, C) n=6-8 mice per treatment group. Data are presented as mean + SEM and and effects of treatment were analysed by unpaired Student’s t-test (*p<0.05, **p<0.01).

**Suppl Fig 1
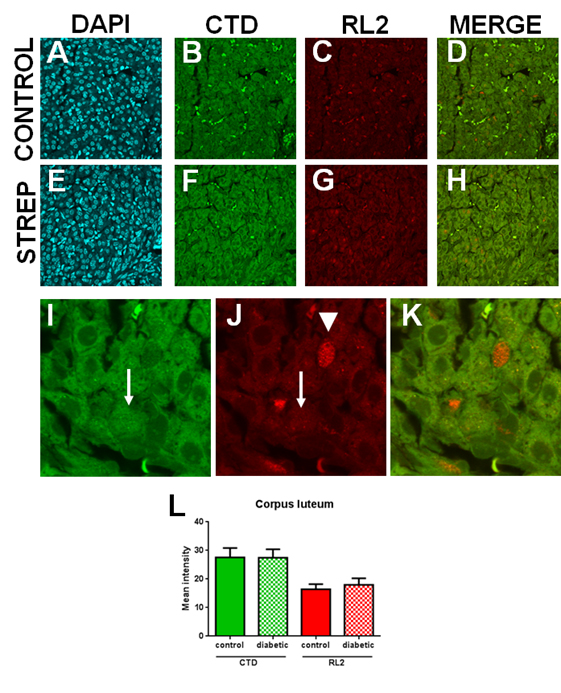
**

**Suppl Fig 2**

**
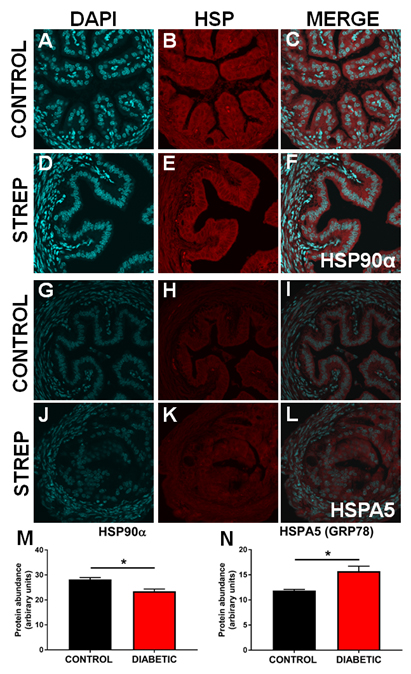
**

**Suppl Fig 3**

**
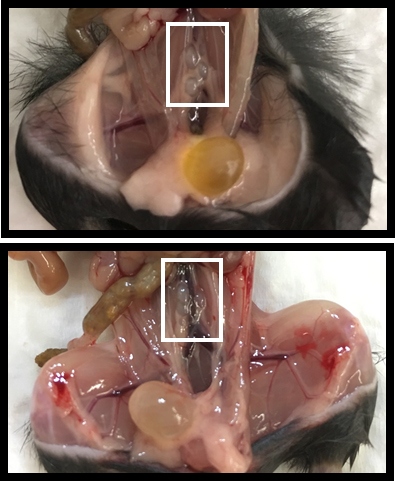
**

**Suppl Fig 4**

**
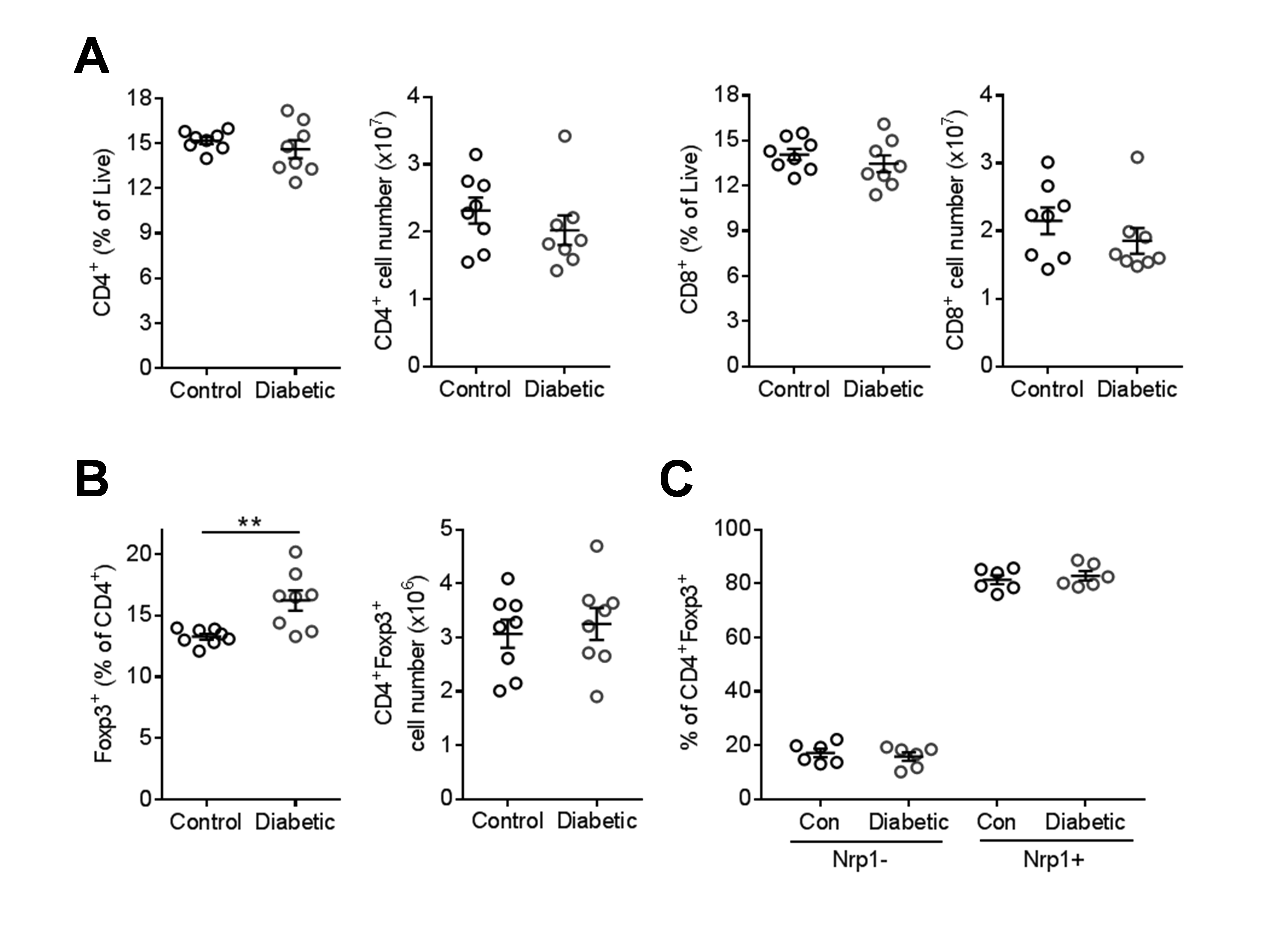
**
